# Plasmid-Mediated Antibiotic Resistant *Escherichia coli* in Sarawak Rivers and Aquaculture Farms, Northwest of Borneo

**DOI:** 10.3390/antibiotics10070776

**Published:** 2021-06-25

**Authors:** Samuel Lihan, Sai Y. Lee, Seng C. Toh, Sui S. Leong

**Affiliations:** 1 Institute of Biodiversity and Environmental Conservation, Universiti Malaysia Sarawak, Kota Samarahan 94300, Malaysia; lsamuel@unimas.my; 2 Faculty of Resource Science and Technology, Universiti Malaysia Sarawak, Kota Samarahan 94300, Malaysia; saiyeng@gamil.com; 3 Department of Animal Sciences and Fishery, Faculty of Agricultural Science and Forestry, Universiti Putra Malaysia, Nyabau Road, Bintulu 97008, Malaysia; kelvintoh0413@gmail.com

**Keywords:** drug resistant, aquaculture, extrachromosomal, *Escherichia coli*, water source, plasmid

## Abstract

Background: The emergence of plasmid-mediated antibiotic resistance in *Escherichia coli* in water resources could pose a serious threat to public health. The study aims to investigate the dispersion of plasmid-mediated antibiotic-resistant *E. coli* from six rivers in Sarawak and two aquaculture farms in Borneo. Methods: A total of 74 water samples were collected for the determination of their bacteria colony count. An IMViC test identified 31 *E. coli* isolates and tested their susceptibility against twelve clinically important antibiotics. The extraction of plasmid DNA was done using alkali lysis SDS procedures. Characteristics, including plasmid copy number, molecular weight size, resistance rate and multiple antibiotic resistance (MAR), were assessed. Results: Our findings revealed that bacterial counts in rivers and aquaculture farms ranged from log 2.00 to 3.68 CFU/mL and log 1.70 to 5.48 cfu/mL, respectively. Resistance to piperacillin (100%) was observed in all *E. coli*; resistance to amoxicillin (100%) and ampicillin (100%) was observed in *E. coli* found in aquaculture farms; resistance to streptomycin (93%) was observed in *E. coli* found in rivers. All *E. coli* were resistant to ≥2 antibiotics and formed 26 MAR profiles, ranging from an index of 0.17 to 0.83, indicating that there are high risks of contamination. Some (48.4%) of the *E. coli* were detected with plasmids (1.2 to >10 kb), whereas 51.6% of the *E. coli* did not harbor any plasmids. The plasmid copy numbers reported were one plasmid (*n* = 7), two plasmids (*n* = 4), ≥ two plasmids (4). *E. coli* isolated from the Muara Tuang River showed the highest-molecular-weight plasmids. A statistical analysis revealed that there is no significant correlation (r = 0.21, *p* = 0.253) between the number of plasmids and the MAR index of the tested isolates. Conclusion: The distribution of MAR in *E. coli* from rivers is higher compared to the aquaculture environment. Our study suggests that MAR in isolates could be chromosome-mediated. Our results suggest that riverbed sediments could serve as reservoirs for MAR bacteria, including pathogens, under different climatic conditions, and their analysis could provide information for public health concerns.

## 1. Introduction

The occurrence of antibiotics in the aquatic environment, especially in aquaculture farms and rivers, has been viewed as one of the major threatening issues causing environmental pollution worldwide. Aquaculture is a fast-growing food-production industry, supplying 30.1 million tons of aquatic plants, 80 million tons of total food fish and 37,900 tons of non-food products [1] to the growing world population. The usage of antimicrobial agents in aquaculture has increased exponentially, accounting for 63,151 tons in 2010, and is expected to increase to 67% by 2030 [2]. Brazil, Russia, Africa and India are the countries recorded with the highest global antimicrobial consumption [3]. Most farmers do not use antibiotics appropriately and responsibly, especially in raising fish and shrimp either in ponds or rivers, thus becoming a risk to end-users. Rivers are contaminated with antibiotics, which are released into the environment through hospital wastewater [4,5,6], sewage influent and effluent [7], sludge [8], surface run-off [7], plantation activities, treatment plants and water-body sediments [9].

Raufu et al. [10] have proved that water resources such as farms and rivers serve as reservoirs or disseminators of resistant bacterial strains, when they investigated the occurrence of antimicrobial-resistant *Salmonella* from fishes in Nigeria. The extensive abuse of antibiotics has caused multiple antibiotic resistance (MAR) in *Escherichia coli* strains, which were then disseminated through the food chain in ecosystems. MAR in *E. coli* strains is routinely associated with extrachromosomal DNA plasmids that consist of one or more resistance genes [11,12]. Plasmids are circular DNA that facilitates the spread of antibiotic-resistant genes from one bacterium to another and pose a threat to human health. Moreover, when Munita and Arias [13] reviewed the mechanism behind bacterial antibiotic resistance, they explained that the large amount of antibiotic-resistance bacteria found in the environment often correlates with resistance-trait plasmids. Antibiotic-resistant genes are commonly transmitted to the animal and human intestinal-tract microflora through virulent pathogens. Researchers believe that most human and farming activities near water resources may increase the prevalence of antibiotic-resistant bacteria [14].

*E. coli* are an opportunistic pathogen that can endure well in aquatic systems and are exceptionally proficient at horizontal gene transfer, which is believed to be the vector for antibiotic-resistance dissemination [15]. Multidrug-resistant *Enterobacteriaceae* are of public concern, especially disease-causing *E. coli* [16], mainly because *E. coli* has been reported to show resistance to all regularly prescribed antibiotics [17]. Subba et al. [18], who investigated the plasmid profiles of thermotolerant *E. coli* in drinking water, has found that antibiotic-resistant *E. coli* can transmit their antibiotic resistance to other water-borne pathogens. Urgent attention is needed to detect plasmid-mediated antibiotic-resistant *E. coli* in order to avoid any outbreak or treatment failure that could lead to the multiplication of virulent *E. coli* in the ecosystem. The full spectrum of the environmental dissemination of plasmid-mediated antibiotic-resistant *E. coli* in water ecosystems is lacking. Hence, this study aims to investigate the distribution of plasmid-mediated antibiotic-resistant *E. coli* from aquaculture farms and rivers and the relationship between the plasmid and antibiotic resistance. The hypothesis is that (1) *E. coli* isolates from different water sources may have different antibiotic resistance levels and that (2) plasmids may relate to the multiple antibiotic resistance (MAR) index.

## 2. Results

### 2.1. Bacteria Colony Count

56 samples were collected from 3 aquaculture farms, and 18 samples were collected from 6 rivers in Sarawak. Mean bacterial counts for *E. coli* ranged from log 2.00 to 3.68 CFU/ mL and log 1.70 to 5.48 CFU/mL, found in rivers and aquaculture farms, respectively, as shown in Table 1. The presence of *E. coli* was significantly (*p* = 0.05) different between the study sites.

### 2.2. Escherichia coli Isolation

A total of 36 bacterial isolates that showed a metallic greenish sheen on EMBA were selected and subjected to morphological and biochemical identification. Biochemical tests, which included Gram staining and an IMViC test, were carried out to obtain a more definitive identification of *E. coli*. The IMViC test result of presumptive *E. coli* is shown in Table 2.

### 2.3. Antibiotic Susceptibility Test

**Figure 1:**
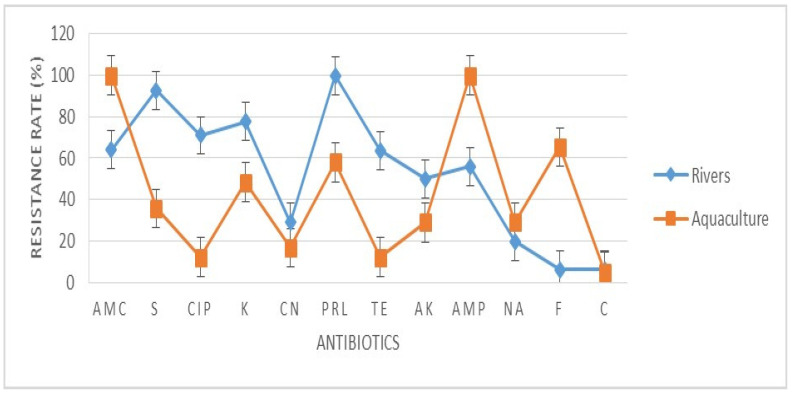
The antibiotic resistance rate (%) of *Escherichia coli* isolated from aquaculture farms and Sarawak Rivers against 12 antibiotics. AMC: amoxicillin, S: streptomycin, CIP: ciprofloxacin, K: kanamycin, CN: gentamicin, PRL: piperacillin, TE: tetracycline, AK: amikacin, AMP: ampicillin, NA: nalidixic acid, F: nitrofurantoin, C: chloramphenicol. Values represent the mean (%) of three replications. Vertical bars indicate the standard error of the means.

**Figure 2:**
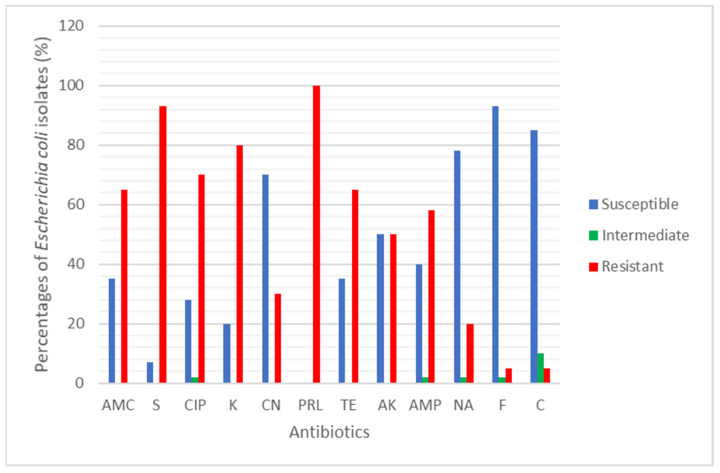
The percentage (%) of *Escherichia coli* isolated from 6 Sarawak rivers that showed Susceptible, Intermediate and Resistant against the 12 antibiotics tested.

**Figure 3:**
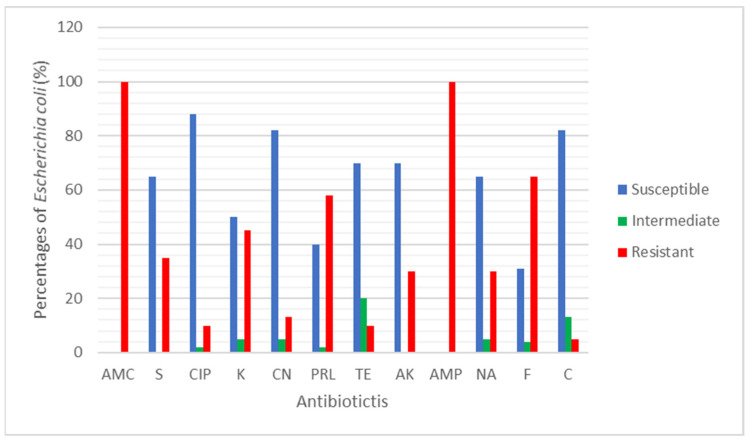
The percentage (%) of *Escherichia coli* isolated from aquaculture farms that showed Susceptible, Intermediate and Resistant against the 12 antibiotics tested.

**Figure 4:**
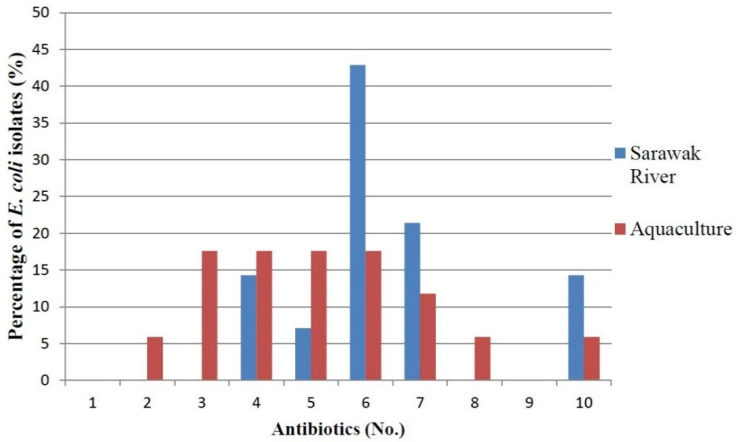
Multiple-antibiotic-resistance profile of *E. coli* from 6 Sarawak rivers and 3 aquaculture farms.

A total of 31 *E. coli* were tested against 12 clinical antibiotics. The antibiotic resistance rate (%) of the *E. coli* found in rivers and aquaculture farms are shown in Figure 1. Figure 2 and Figure 3 showed the frequency (%) of *E. coli* in rivers and aquaculture farms that displayed sensitivity, intermediate resistance and resistance to the antibiotic tested, respectively. Resistance to amoxicillin and ampicillin was observed in 100% of *E. coli* found in aquaculture farms, while resistance to streptomycin (93%) and piperacillin (100%) was observed in *E. coli* found in rivers. The overall resistance rate of *E. coli* found in rivers was higher compared *E. coli* found in aquaculture farms (Figure 1). The results revealed that most of the *E. coli* found in rivers were susceptible to gentamicin (72%), nalidixic acid (78%), nitrofurantoin (93%) and chloramphenicol (85%) (Figure 2), while *E. coli* found in aquaculture farms were susceptible to ciprofloxacin (88%), gentamicin (82%) and chloramphenicol (82%) (Figure 3). Figure 4 shows the multiple-antibiotic-resistance profile of *E. coli*, demonstrating that most of the *E. coli* found in rivers were highly resistant to at least 4 to 10 antibiotics, with resistance to 6 antibiotics (43%) being the highest percentage recorded. *E. coli* found in aquaculture farms were resistant to at least 2 to 10 antibiotics.

### 2.4. Plasmid Detection and Size Estimation

**Figure 5:**
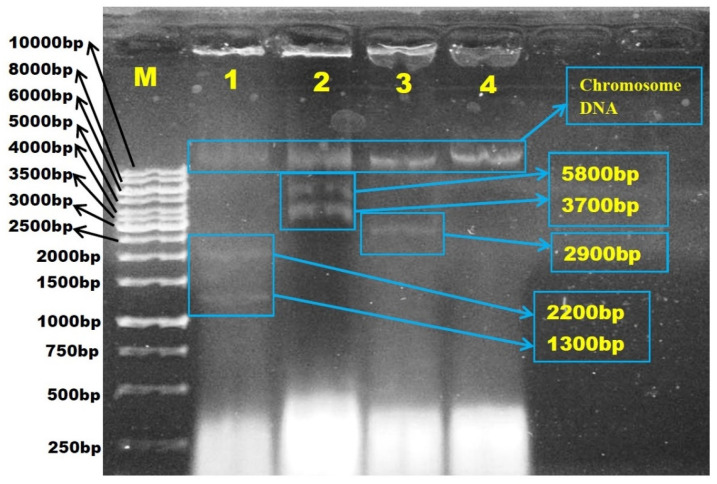
Agarose gel electrophoresis photograph of the estimated molecular weight size (kb) of the plasmid fragments in *Escherichia coli* isolated from Sarawak rivers and aquaculture farms. The estimated plasmid size was measured by comparing their band patterns obtained in agarose gel with linear DNA markers. Lane M, 1kb ladder; Lane 1, *E. coli* ATCC 25922; Lane 2, WF-S2B; Lane 3, WF-S3B, Lane 4, BT-S3.

**Figure 6:**
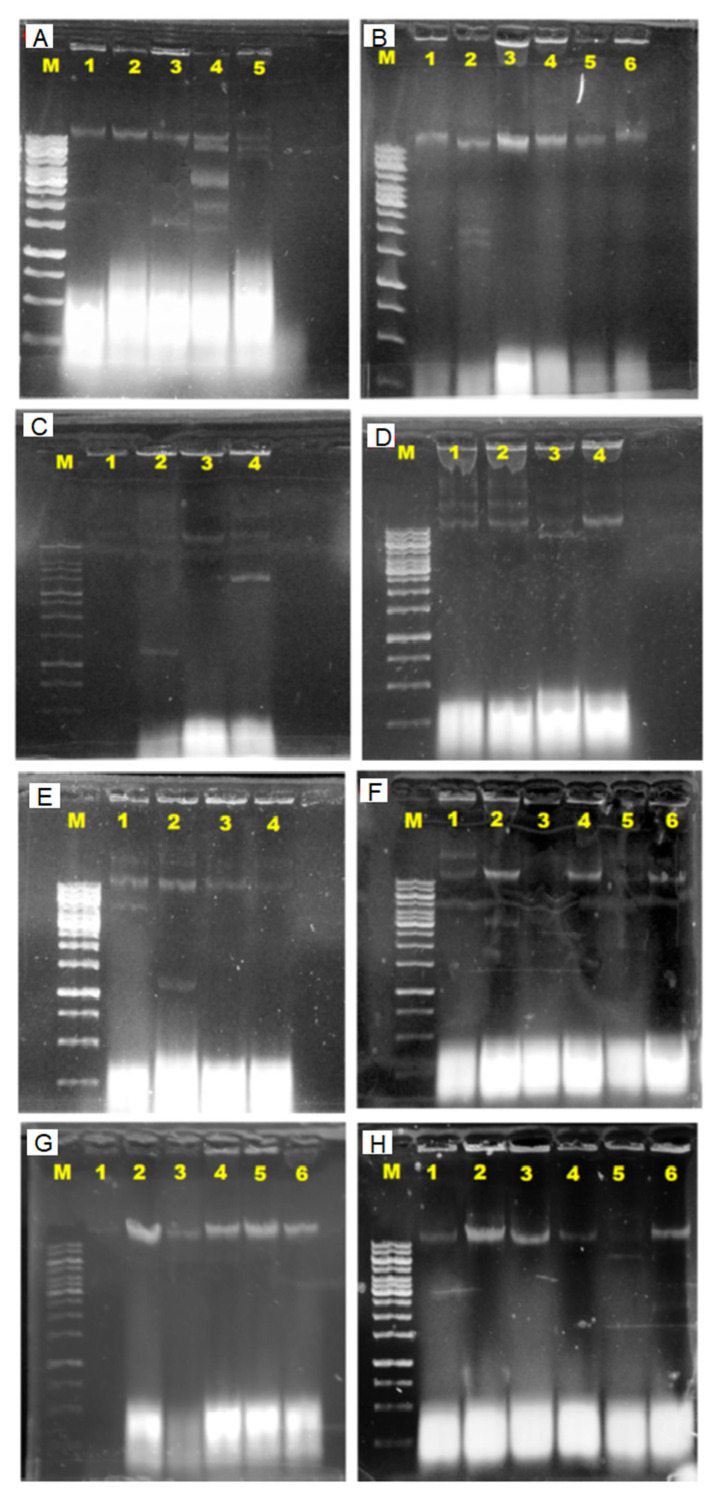
Agarose gel electrophoresis photograph of the estimated molecular weight (1.1- >10 kb) of the plasmid fragments in *Escherichia coli* isolated from Sarawak rivers and aquaculture farms. The plasmid DNA band shown above the chromosomal DNA band was estimated to carry >10 kb molecular weight. Lane M, 1kb ladder; *E. coli* isolates (**A**) Lane 1, *E. coli* ATCC 25922; Lane 2, WC-S1A; Lane 3, SN-S2B; Lane 4, MT-S1A; Lane 5, SN-S1B; (**B**) Lane 1, AS-62(S); Lane 2, BK-RV1(S); Lane 3, AS-8(S); Lane 4, AS-NS(S)2; Lane 5, BK2-K4(S); Lane 6, AS-16(S); (**C**) Lane 1, AS-SD2(B); Lane 2, BK2-OLT(B); Lane 3, AS-16(B); Lane 4, BK2-OLT(S); (**D**) Lane 1, AS-R10(S); Lane 2, AS-62(B); Lane 3, BK2-K2(B); Lane 4, AS-R10(B); (**E**) Lane 1, BK2-OLT(S); Lane 2, BK2-OLT(B); Lane 3, AS-16(B); Lane 4, BK-RV2(S); (**F**) Lane 1, BT-S3B; Lane 2, WF-S3B; Lane 3, WF-S2B; Lane 4, SN-S2B; Lane 5, BK-RV2(S); Lane 6, SN-S1B; (**G**) Lane 1, WF-S3 B; Lane 2, WC-S3A P; Lane 3, WF-S1B; Lane 4, SN-S1B; Lane 5, AS-42(S); Lane 6, BT-S1B; (**H**) Lane 1, SN-S1A; Lane 2, AS-62(B); Lane 3, AS-R10(B); Lane 4, AS-16(B); Lane 5, WF-S1B; Lane 6, BK2-K2(B).

In order to investigate the involvement of plasmids in antibiotic resistance, plasmid DNA was extracted from all 31 isolates that exhibited resistance to at least one antibiotic (Figure 4). Figure 5 demonstrated the estimated plasmid fragments’ molecular weight by comparing their band patterns obtained in agarose gel with linear DNA markers. The chromosomal DNA band was shown as a crown-shaped band at the same line in gel, and the plasmid DNA bands were shown as bands above or below the chromosomal DNA band, depending on their size. In the study, plasmids of various numbers and sizes were found. *E. coli* carried from one to six plasmids, and estimated sizes ranging from about 1.1- >10 kb are shown (Figure 6).

### 2.5. Analysis of Plasmid Size and Antibiotic Resistance

MAR patterns and plasmid size of the total 31 *E. coli* isolates are documented in Table 3. All *E. coli* were resistant to at least two or more antibiotics and formed 26 MAR profiles, ranging from 0.17 to 0.83, indicating high-risk sources of contamination. There were 15 isolates that harbored one or more plasmids with molecular weight between 1.1 and >10 kb, estimated through plasmid DNA gel analysis. The plasmid profiling documented in Table 3 reported that 48.4% of the *E. coli* from both sources harbor plasmids (1.2 to >10 kb size), whereas 51.6% of the *E. coli* did not consist any plasmid. There were seven *E. coli* detected with a single plasmid, four detected with two plasmids and four detected with more than two plasmids. A portion (73.3%) of the high-molecular weight-plasmids are reported in Table 3. However, most *E. coli* that were resistant to more than two antibiotics tested without any plasmid detected. The *E. coli* isolated from the Muara Tuang River was detected with the highest-molecular weight-plasmids (1.1, 1.5, 2.6, 3.7 and >10.0 kb). Furthermore, the abundance of plasmid occurrence was 50% in rivers and 78% found in aquaculture farms. A statistical analysis revealed that there was no significant correlation (r = 0.21, *p* = 0.253) between the number of plasmids and the MAR index of the tested isolates.

## 3. Discussion

A total of 74 water samples were collected from rivers and aquaculture farms, with 31 *E. coli* being successfully identified by Gram staining and an IMViC test. *E. coli* is a fecal coliform and an indicator of fecal contamination in water sources. The prevalence of antibiotic-resistant *E. coli* found in water resources [19] has become so widespread in most developing countries that we can no longer ignore it. Our findings have disclosed that most of the *E. coli* found in aquaculture farms were totally (100%) resistant to amoxicillin and ampicillin (Figure 1). Most of the *E. coli* were resistant to at least two (3.2%) or more (96.8%) antibiotics tested (Figure 4). Researchers believe that extensive use of various antibiotics in aquaculture farming directly or indirectly along the riverbank may lead to the emergence of multiple antibiotic resistance in bacteria [20,21]. Our findings showed that the resistance of *E. coli* to ampicillin and amoxicillin, part of the first- and second-generation cephalosporin antibiotic group, was widespread. Our findings were concurrent with research done by Wong et al. [22] analyzing urine samples in Hong Kong, which showed that most *E. coli* had developed resistance to ampicillin. However, Wong et al. [22] reported that the *E. coli* isolated were susceptible to amoxicillin, which contradicts our findings. Amoxicillin is the most common clinically prescribed antibiotic for patients suffering from respiratory and urinary-tract infections. Resistance to ampicillin and amoxicillin in Gram-negative bacteria is not something new, as it corresponds to similar findings obtained by other researchers in several other countries [23,24,25]. Apparently, countries that practice aquaculture farming, such as Malaysia, still utilize antibiotics for therapeutic or prophylactic purposes. Unfortunately, these aquaculture farming systems have caused antibiotic residue to build up in the water and in fish tissue, plankton and even in sediments in the ponds, all of which indirectly affect our food safety and ecosystem. Our findings reveal that most *E. coli* found in rivers were highly resistant to piperacillin (100%) and streptomycin (93%) (Figure 1). Most of the environmental *E. coli* were highly resistant to piperacillin and streptomycin, as reported previously in clinical samples [26,27], birds [28] and the environment [29] but not in water samples. Streptomycin is an antibiotic widely used in agriculture to promote animal growth [30]. Chikwendu et al. [31] reported similar findings, that moderate resistance to ampicillin is detectable in *E. coli* found in rivers. Thus, the uncontrolled use of these antibiotics may drain into rivers and could cause a terrifying scenario.

Our findings revealed that chloramphenicol was among the most susceptible antibiotics. Low resistance toward chloramphenicol was mainly because it has been banned for use in livestock since 1982 [32]. Our result showed that most *E. coli* isolated from aquaculture farms were susceptible to ciprofloxacin and tetracycline. According to Christabel et al. [33], ciprofloxacin is the most effective treatment in 99% of typhoid cases. Our results correlated with the result conducted by Shahriar and Khair [34]. In addition, low resistance of *E. coli* isolates from aquaculture against nalidixic acid was similar to previous studies suggesting that this antibiotic was not registered for use in livestock [24]. The high antibiotic resistance reported in our study may be caused by contamination. *E. coli* isolated from rivers and aquaculture farms were reported to be resistant to more than one class of antibiotic. Multiple drug resistance has been previously documented in the studies of marine pathogens and aquaculture environments [23,35,36,37]. In general, our findings showed 26 MAR patterns against the 12 antibiotics tested in this present study. The MAR ranged from 0.17 to 0.83 (Table 3), which indicates that most *E. coli* originates from high-risk sources of contamination [38]. This is possibly due to the water samples being collected from areas near the cities and industrial areas where antibiotics were frequently used. *E. coli* isolated from drinking water [39] have demonstrated MAR index >0.2, and resistance to six to ten antibiotics was common. Abia et al. [40], who isolated *E. coli* in rivers in South Africa, reported that over 80% of the *E. coli* isolates were highly resistant to nitrofurantoin and ampicillin, with a high MAR index (≥3 antibiotics). Furthermore, the bacteria isolated from aquaculture farms expressed higher levels of multiple antibiotic resistance, which agreed with previous studies conducted in different water sources in Nigeria [31]. According to Alhaj et al. [37], the high levels of antibiotic-resistant bacteria found in rivers were influenced by human and agricultural activity, and 53.6% of coliform isolates from the Sumjin River in Korea showed resistance to one or more antibiotics. Leong et al. [28] assessed the biorisk of birdhouses in Malaysia and stated that *E. coli* were resistant to up to 10 antibiotics. In addition, the river isolates exhibited high resistance rates due to the availability of nutrients and protection against sunlight in the sediments of rivers. The correlation between MAR levels and the abusive usage of antibiotics was proven by Tendencia and de la Pena [41]. Our findings indicate the use of more than one antibiotic in aquaculture farming, allowing selection pressure for multi-resistant *E. coli* strains. We predict that the occurrence of high-antibiotic-resistant *E. coli* found in rivers may be due to the effluent drained from clinical samples from a nearby hospital. Rivers receive raw sewage from academic and health institutions situated near the riverbank. This waste might be the source of resistant clinical isolates; it flows into the river and causes the spread of resistant traits to other bacteria.

Nearly half of the isolates were found to carry different-sized plasmids, and over 73% of isolates detected with high-molecular-weight plasmids. That the presence of high-molecular-weight plasmids is common in animal farms [42,43], aquaculture and rivers is well-documented [44,45]. Manjusha and Sarita [46] stated that plasmids play an important role in spreading and transferring antibiotic resistance among bacteria. We believe that the transferable R-plasmids are responsible for various antimicrobial resistance associated with water resources, as reported in [13,47,48]. There is a relationship between antibiotic resistance and plasmid size. The larger sizes of plasmids involved in the conjugation process thus enable the dissemination of antibiotic-resistant genes to humans and threaten human health [38]. Besides, the multiple antibiotic resistance in isolates, which do not consist of plasmids, indicate that the antibiotic resistance that arises is due to chromosomes and transposons that aid in the rapid spread of resistance genes to other bacteria [38]. Most plasmid-related studies only focus on tetracycline, chloramphenicol and streptomycin resistance; thus, more research should be done regarding ampicillin, amoxicillin, piperacillin and streptomycin. Antibiotic resistance in medically important bacteria such as *E. coli* is a major concern due to several significant illnesses caused. Markley et al. [49] reported that the alteration of the structure, function and inhibition of these antibiotic-resistance-causing proteins could rescue antibiotic efficacy in clinical or environment.

A statistical analysis revealed that there were no significant statistical differences (r = 0.21, *p* = 0.253) between the number of plasmids and the multiple-antibiotic-resistance (MAR) index of the tested isolates in this study. From the results observed, this suggests that multiple antibiotic resistance in isolates could be of chromosomal origin, and the resistance genes of the bacteria may carry on other extrachromosomal DNA, such as R-plasmids (~50 to ~100 kb) or phage-plasmids. This is proven by the chromosomal DNA band shown on the standard gel (Figure 6) and would be indistinguishable from chromosomal DNA. Our result is in concurrence with previous findings that chromosomes in *E. coli* confer resistance to more than one antibiotic [50]. According to Thavasi et al. [51], the genes encode an antibiotic-resistance phenotype that can be located on transposons and chromosome. According to Sengupta and Austin [52], large plasmids are often present in low copy numbers, and this is similar in the present study that found that most of the plasmids found in *E. coli* isolates from rivers and aquaculture were of high molecular weight. Thus, our study revealed that plasmid numbers and MAR did not correspond with plasmid profiles. Our study alone is inadequate to prove that there is no correlation between the two variables; thus, further research is suggested in the future.

## 4. Materials and Methods

### 4.1. Sampling Areas

**Figure 7:**
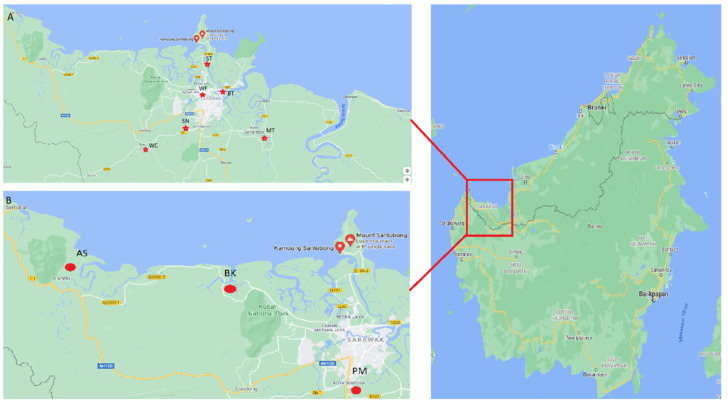
Locations of the study areas in Sarawak, Malaysia, northwestern Borneo (shown in the red box). (**A**) Locations of six sampling stations of rivers and (**B**) locations of three aquaculture farms.

The study was conducted on three aquaculture farms and in six rivers in Sarawak, Malaysia, in the northwest of Borneo. The sources were PM: PM Aquaculture Farms (1°28′12.9 N, 110°19′51.5 E), AS: Asia Aquaculture Farms (1°36′01.2 N, 110°24′29.2 E), BK: Bako Aquaculture Farms (1°36′01.0 N, 110°24′29.5 E); BT: Bintawa River (1°35′49.5 N, 110°22′30.8 E), MT: Muara Tuang River (1°29′42.7 N, 110°23′41.3 E), ST: Santubong River (1°39′32.0 N, 110°21′00.9 E), SN: Siniawan River (1°26′48.3 N, 110°13′10.9 E), WF: Waterfront River (1°33′41.1 N, 110°23′58.9 E) and WC: Windcave River (1°24′52.8 N, 110°08′15.2 E) (Figure 7). Sarawak, a state in Malaysia, stretches along the northwest coastal area of Borneo. It is one of the major oil, gas and agricultural-plantation states in Malaysia. The climate is tropical with alternating rainy (October to March) and dry seasons (April to September). Monthly rainfall is recorded as 374–458 mm duringrainy seasons. The water bodies receive residential waste, along with year-round runoff from the plantations.

### 4.2. Sample Collection

Triplicated water samples were collected from various ponds in aquaculture farms and six rivers, from April to September 2019 using sterile 500 mL falcon tubes. The samples were collected from surface water and from the bottom of each source and placed inside an icebox to avoid adverse composition change in total bacterial count before further analysis in the laboratory.

### 4.3. Bacteria Colony Count and Isolation of Escherichia coli

The water samples were serial diluted, and 20 µL of the mixture was plated on eosin-methylene blue agar (EMBA) (Oxoid, North Shore England) and incubated for 24–48 h at 28 °C. Only bacterial colonies with green metallic colors were subcultured on new EMBA agar to obtain a pure colony. The CFU (CFU/mL) was calculated with the number of colonies per plate multiplied by the dilution factor [53]. The presumptive *E. coli* was further confirmed with indole, methyl-red, Voges-Proskauer and citrate-utilization tests (IMViC), which involved indole, methyl red, Voges–Proskauer, citrate, motility and hydrogen sulfite gas production. The IMViC test is commonly used in differentiating coliform bacteria such as *Escherichia coli*, *Enterobacter aerogenes*, *Enterobacter cloacae* and *Klebsiella pneumoniae* in the *Enterobacteriaceae* family.

### 4.4. Antibiotic-Susceptibility Testing

The antibiotic-sensitivity profiles of the *E. coli* isolates were measured by the standard disc diffusion method as described by the Clinical and Laboratory Standards Institute [54]. A reference culture (*Escherichia coli* ATCC 25922) was used as quality control. A total of 12 types of antibiotics (Oxoid, North Shore, England), including amikacin (30 g), amoxicillin (3 g), ampicillin (2 g), chloramphenicol (30 g), ciprofloxacin (5 g), gentamycin (10 g), kanamycin (30 g), nalidixic acid (30 g), nitrofurantoin (300 g), piperacillin (75 g), streptomycin (10 g) and tetracycline (30 g) were used in this study. The selection of antibiotics was based on their frequent usage in clinical practice and according to the Clinical and Laboratory Standards Institute (CLSI) M45 guideline for *Enterobacteriaceae*. Disk susceptibility testing was adopted from Sien et al. [55]. Overnight *E. coli* suspension (0.5 MacFarland) was inoculated onto the surface of Mueller Hinton (Merck, Darmstadt, Germany) agar and allowed to dry for 5 min. An antibiotic disc was placed onto the agar and incubated at 37 °C for 24 h. The multiple-antibiotic-resistance (MAR) index of *E. coli* was calculated according to Leong et al. [28] to assess the potential health risk. A value ≥ 0.2 indicates MAR, and the number of the MAR index indicates the sensitivity and resistance of *E. coli* to antibiotics.

### 4.5. Plasmid DNA Extraction

The plasmid DNA of *E. coli* strains was extracted by alkali lysis methods as described, with modifications [56]. A single colony of *E. coli* was grown overnight in 10 mL Luria–Bertani (LB) broth (Merck, Darmstadt Germany) in an orbital shaker for 16–18 h. A portion (2 mL) of the overnight culture was centrifuged at 8000 rpm for 2 min in a microcentrifuge tube. This step was repeated twice until a pellet was obtained. The supernatant was discarded, and the pellet was resuspended in 100 µL of solution I. Next, the tubes were vortexed for 10 s and kept on ice. An amount (100 µL) of solution II was added, and the tubes were inverted 10× gently. Microcentrifuge tubes were left at room temperature for 5 min. A clear viscous liquid was observed. After that, 300 µL of solution III was added, and the tubes were inverted 10× gently. A white precipitate was observed, and the tubes were centrifuged at 10,000 rpm for 5 min. Then, the supernatant was transferred to a new sterile tube, and 125 µL of cold absolute ethanol were added. Tubes were inverted 10× gently before being centrifuged at 13,000 rpm for 5 min. After that, the supernatant was discarded, and 500 µL of 70% ethanol was added, followed by centrifuging at 13,000 rpm for 2 min. The pellet was then left to air-dry for 15 min, after which the supernatant was discarded before it was resuspended in 50 µL of sterile ultrapure water. The pellet was subjected to agarose gel electrophoresis.

### 4.6. Viewing the Plasmid Product

Plasmid products were loaded on the gel by mixing 40 µL of the resuspended pellet with 5 µL of loading dye (Promega, Madison, WI, USA). Then, plasmid products were analyzed by electrophoresis in 0.8% (*w*/*v*) agarose stained with 10 µL of 0.5 μg/mL of ethidium bromide in 1× TBE buffer at 100 V and 200 mA for 55 min. A 1 kb DNA ladder (Promega, Madison, WI, USA) was used as a linear DNA marker. Plasmid fragments were then visualized with UV transilluminator. The estimated molecular weight (kb) of the plasmid fragments were measured by comparing their band pattern obtained in agarose gel electrophoresis with the DNA marker.

### 4.7. Statistical Analysis

Statistically significant differences between the plasmid copy number and MAR were assessed by using one-way ANOVA methods (SPSS, version 20, IBM, New York, NY, USA) at a significance level of *p* < 0.05.

## 5. Conclusions

In this study, the distribution of multiple antibiotic resistance in *E. coli* from rivers are higher compared to those from aquaculture. This might be caused by the existence of commercial industry and health institutions that frequently use antibiotics and are located near rivers. Therefore, the rational use of antibiotics is essential to reducing the acquisition of antibiotic resistance in both river and aquaculture bacteria. All the *E. coli* isolated from aquaculture tested in the present study were resistant to amoxicillin and ampicillin, whereas all the *E. coli* isolated from rivers were resistant to piperacillin and streptomycin. Moreover, plasmid profiling revealed that 48.4% of the antibiotic-resistance *E. coli* from both aquaculture and rivers possess one to six plasmids in which their size ranged from 1.1 to >10 kb and 51.6% and did not reveal any plasmid DNA. A statistical analysis revealed that there were no significant statistical differences (r = 0.21, *p* = 0.253) between the number of plasmids and the multiple-antibiotic-resistance (MAR) index of the tested isolates, and this suggests that multiple antibiotic resistances in isolates could be of chromosomal origin and that some of the antibiotic-resistance in bacteria was chromosome-mediated. Our results suggest that riverbed sediments could serve as reservoirs for MAR bacteria, including pathogens, under different climatic conditions, and their analysis could provide information for public health concerns.

## Figures and Tables

**Table 1:** Mean bacteria colony count (log CFU/mL) of water samples collected from Sarawak rivers and aquaculture farms in Sarawak, Malaysia and northwest of Borneo.

Sites	Samples	Source	Mean Bacteria Colony Count (CFU/mL)	Mean Bacteria Colony Count (log CFU/mL)
PM	PM-W1	Pond water	1.10 × 10^4^	4.04
PM	PM-W2	Pond water	1.18 × 10^4^	4.07
PM	PM-W3	Pond water	1.80 × 10^3^	3.26
PM	PM-W4	Pond water	6.90 × 10^3^	3.84
PM	PM-W5	Pond water	4.00 × 10^2^	2.60
PM	PM-W6	Pond water	3.70 × 10^3^	3.57
PM	PM-W7	Pond water	2.40 × 10^3^	3.38
PM	PM-W8	Pond water	8.50 × 10^3^	3.93
PM	PM-W9	Pond water	1.90 × 10^3^	3.28
PM	PM-W10	Pond water	3.00 × 10^5^	5.48
PM	PM-W11	Pond water	3.00 × 10^5^	5.48
AS	AS-NS(S)1	Shrimp hatcheries, surface water	5.00 × 10^1^	1.70
AS	AS-NS(S)2	Shrimp hatcheries, surface water	1.50 × 10^3^	3.18
AS	AS-R10(S)	Reservoir, surface water	5.50 × 10^3^	3.74
AS	AS-R10(B)	Reservoir, Deep water	1.50 × 10^2^	2.18
AS	AS-42(S)	Surface water	2.00 × 10^3^	3.30
AS	AS-42(B)	Deep water	5.00 × 10^1^	1.70
AS	AS-62(S)	Surface water	4.50 × 10^3^	3.65
AS	AS-62(B)	Deep water	5.00 × 10^1^	1.70
AS	AS-SD1(S)	sediment	4.00 × 10^3^	3.60
AS	AS-SD1(B)	sediment	3.00 × 10^5^	5.48
AS	AS-26(S)	Surface water	4.15 × 10^3^	3.62
AS	AS-26(B)	Deep water	7.00 × 10^2^	2.85
AS	AS-16(S)	Surface water	2.00 × 10^3^	2.30
AS	AS-16(B)	bottom of pond	1.50 × 10^3^	3.18
AS	AS-SD2(S)	sediment	7.50 × 10^3^	3.88
AS	AS-SD2(B)	sediment	4.30 × 10^3^	3.63
AS	AS-8(S)	Surface water	1.10 × 10^3^	3.04
AS	AS-8(B)	Deep water	5.00 × 10^1^	1.70
BK	BK-K1(S)	Surface water, pond	5.00 × 10^1^	1.70
BK	BK-K1(B)	Deep water, pond	2.50 × 10^2^	2.40
BK	BK-K2(S)	Surface water, pond	5.00 × 10^1^	1.70
BK	BK-K2(B)	Deep water, pond	5.00 × 10^1^	1.70
BK	BK-K6(S)	Surface water, pond	5.00 × 10^1^	1.70
BK	BK-K6(B)	Deep water, pond	5.00 × 10^1^	1.70
BK	BK-K7(S)	Surface water, pond	5.00 × 10^1^	1.70
BK	BK-K7(B)	Deep water, pond	3.00 × 10^2^	2.48
BK	BK-K8(S)	Surface water, pond	6.90 × 10^3^	3.84
BK	BK-K8(B)	Deep water, pond	3.00 × 10^5^	5.48
BK	BK-RV1(S)	Reservoir, surface water	6.50 × 10^2^	2.81
BK	BK-RV2(S)	Reservoir, surface water	2.00 × 10^2^	2.30
BK	BK-ST1(S)	Stagnant pond, Surface water	4.00 × 10^3^	3.60
BK	BK-ST2(B)	Stagnant pond, Deep water	3.00 × 10^5^	5.48
BK	BK-RS1(S)	Reservoir, surface water	6.65 × 10^3^	3.82
BK	BK-RS2(S)	Reservoir, surface water	1.29 × 10^4^	4.11
BK	BK2-K1(S)	Surface water, pond	5.00 × 10^1^	1.70
BK	BK2-K1(B)	Deep water, pond	5.00 × 10^1^	1.70
BK	BK2-K2(S)	Surface water, pond	6.25 × 10^3^	3.80
BK	BK2-K2(B)	Deep water, pond	3.00 × 10^5^	5.48
BK	BK2-RS(S)	Reservoir, Surface water	3.00 × 10^5^	5.48
BK	BK2-K4(S)	Surface water, pond	3.90 × 10^3^	3.59
BK	BK2-K4(B)	Deep water, pond	3.50 × 10^3^	3.54
BK	BK2-OLT(S)	Outlet pond, surface water	3.00 × 10^5^	5.48
BK	BK2-OLT(B)	Outlet pond, deep water	2.60 × 10^3^	3.41
BK	BK2-TRM(S)	Treatment pond, surface water	5.00 × 10^1^	1.70
BK	BK2-TRM(B)	Treatment pond, deep water	5.00 × 10^1^	1.70
BT	BT-S1	Midstream	4.80 × 10^3^	3.68
BT	BT-S2	Midstream	4.50 × 10^3^	3.65
BT	BT-S3	Midstream	1.10 × 10^3^	3.04
SN	SN-S1	Midstream	2.00 × 10^3^	3.30
SN	SN-S2	Midstream	1.35 × 10^3^	3.13
SN	SN-S3	Midstream	1.50 × 10^3^	3.18
WF	WF-S1	Midstream	2.55 × 10^3^	3.41
WF	WF-S2	Midstream	2.20 × 10^3^	3.34
WF	WF-S3	Midstream	2.20 × 10^3^	3.34
ST	ST-S1	Midstream	5.00 × 10^2^	2.70
ST	ST-S2	Midstream	1.00 × 10^2^	2.00
ST	ST-S3	Midstream	1.50 × 10^2^	2.18
WC	WC-S1	Midstream	1.45 × 10^3^	3.16
WC	WC-S2	Midstream	6.50 × 10^2^	2.81
WC	WC-S3	Midstream	6.50 × 10^2^	2.81
MT	MT-S1	Midstream	7.50 × 10^2^	2.88
MT	MT-S2	Midstream	1.90 × 10^3^	3.28
MT	MT-S3	Midstream	1.60 × 10^3^	3.20

Legend: PM: PM Aquaculture Farms; AS: Asia Aquaculture Farms; BK: Bako Aquaculture Farms; BT: Bintawa River; MT: Muara Tuang River; ST: Santubong River; SN: Siniawan River; WF: Waterfront River; WC: Windcave River.

**Table 2:** IMViC test result of presumptive *E. coli* isolated from 3 aquaculture farms and 6 Sarawak rivers in the northwest of Borneo.

Samples	Indole	Motility	H_2_S Gas Production	MR	VP	CA
Control	−	+	+	+	−	−
PM-W1	−	+	+	+	−	−
PM-W8	−	+	+	+	−	−
PM-W10	−	+	+	+	−	−
AS-RV1(S)	+	+	−	+	−	−
AS-NS(S)2	+	+	−	+	−	−
AS-R10(S)	+	+	−	+	−	−
AS-R10(B)	+	+	−	+	−	−
AS-42(S)	+	+	−	+	−	−
AS-62(S)	+	+	−	+	−	−
AS-62(B)	+	+	−	+	−	−
AS-SD1(S)	+	+	−	+	−	−
AS-16(S)	+	+	−	+	−	−
AS-16(B)	+	+	−	+	−	−
AS-SD2(B)	+	+	−	+	−	−
AS-8(S)	+	+	−	+	−	−
BK-RV2(S)	+	+	−	+	−	−
BK2-K2(B)	+	+	−	+	−	−
BK2-K4(S)	+	+	−	+	−	−
BK2-OLT(S)	+	+	−	+	−	−
BK2-OLT(B)	+	+	−	+	−	−
BT-S1B	+	+	−	+	−	−
BT-S2B	−	+	−	+	−	−
BT-S3B	+	+	−	+	−	−
SN-S1ASN-S1B	+	+	−	+	−	−
SN-S2B	+	+	−	+	−	−
WF-S1B	+	+	−	+	−	−
WF-S2B	+	+	−	+	−	−
WF-S3A	+	+	−	+	−	−
WF-S3B	+	+	−	+	−	−
WC-S1A	+	+	−	+	−	−
WC-S3A	+	+	−	+	−	−
WC-S3B	+	+	−	+	−	−
MT-S1AMT-S1B	+	+	−	+	−	−
MT-S2B	−	−	+	+	−	−

Legend: Control: *Escherichia coli* ATCC 25922; morphology: rod, negative; MR: methyl red; VP: Voges–Proskauer; CA: citrate test; H_2_S: hydrogen sulfite; + positive result; − negative result.

**Table 3:** Multiple-antibiotic-resistance (MAR) patterns and plasmid profiling of *E. coli* isolated from 6 Sarawak rivers and 3 aquaculture farms in Sarawak in northwestern Borneo.

Pattern	Code	Resistance to Number of Antibiotics	Antibiotic Resistance Profile	MAR Index	Number ofPlasmids	Plasmid Size (kb)
1	WF-S1B	6	AK, AMC, K, PRL, S, TE	0.50	1	5.80
2	WF-S2B	6	AK, CIP, K, PRL, S, TE	0.50	2	3.70, 5.80
2	WF-S3A	6	AK, CIP, K, PRL, S, TE	0.50	0	None detected
2	BT-S3B	6	AK, CIP, K, PRL, S, TE	0.50	0	None detected
3	MT-S1B	4	AMC, AMP, PRL, S	0.33	0	None detected
3	WC-S3A	4	AMC, AMP, PRL, S	0.33	0	None detected
4	WF-S3B	6	AK, CIP, K, NA, PRL, TE	0.50	1	2.90
5	BT-S1B	7	AK CIP, CN, K, PRL, S, TE	0.58	0	None detected
6	SN-S1B	10	AMC, AMP, C, CIP, CN, K, NA, PRL, S, TE	0.83	3	3.20, 3.70, 5.80
7	SN-S1A	5	AMC, AMP, CIP, PRL, S	0.42	0	None detected
8	SN-S2B	10	AK, AMC, AMP, CN, F, K, NA, PRL, S, TE	0.83	1	1.15
8	AS-16(B)	10	AK, AMC, AMP, CN, F, K, NA, PRL, S, TE	0.83	1	>10.0
9	MT-S1A	6	AMC, AMP, CIP, K, PRL, S	0.50	6	1.10, 1.50, 2.60, 3.70, >10.0, >10.0,
10	WC-S1A	7	AMC, AMP, CIP, CN, K, PRL, S	0.58	0	None detected
11	WC-S3B	7	AMC, AMP, CIP, K, PRL, S, TE	0.58	0	None detected
12	AS-R10(S)	5	AMC, AMP, F, K, PRL	0.42	2	>10.0, >10.0
13	AS-SD1(S)	4	AMC, AMP, F, PRL	0.33	0	None detected
13	AS-42(S)	4	AMC, AMP, F, PRL	0.33	0	None detected
14	AS-SD2(B)	3	AMC, AMP, S	0.25	0	None detected
15	BK-RV1(S)	5	AK, AMC, AMP, PRL, S	0.42	2	1.30, 1.65
16	BK-RV2(S)	6	AMC, AMP, F, K, S, TE	0.50	1	>10.0
17	BK2-K2(B)	7	AMC, AMP, CIP, K, NA, PRL, S	0.58	1	>10.0
18	BK2-K4(S)	6	AK, AMC, AMP, F, K, PRL	0.50	0	None detected
19	BK2-OLT(S)	6	AK, AMC, AMP, F, K, NA	0.50	2	5.00, >10.0
20	BK2-OLT(B)	8	AK, AMC, AMP, C, CIP, F, NA, TE	0.67	2	1.15, >10.0
21	AS-NS(S)2	2	AMC, AMP	0.17	0	None detected
22	AS-R10(B)	3	AMC, AMP, F	0.25	2	>10.0, >10.0
23	AS-62(S)	5	AMC, AMP, CN, F, PRL	0.42	0	None detected
24	AS-62(B)	3	AMC, AMP, PRL	0.25	2	>10.0, >10.0
25	AS-16(S)	7	AMC, AMP, F, K, NA, PRL, S	0.58	0	None detected
26	AS-8(S)	4	AMC, AMP, CN, K	0.33	0	None detected
27	*E. coli ATCC 25922*	0	-	0	2	1.30, 2.20

Legend: Antibiotics tested: AMC: amoxicillin, S: streptomycin, CIP: ciprofloxacin, K: kanamycin, CN: gentamicin, PRL: piperacillin, TE: tetracycline, AK: amikacin, AMP: ampicillin, NA: nalidixic acid, F: nitrofurantoin, C: chloramphenicol.

## Data Availability

Data is contained within the article.
